# An Interplay Between Post-Traumatic Epilepsy and Associated Cognitive Decline: A Systematic Review

**DOI:** 10.3389/fneur.2022.827571

**Published:** 2022-02-24

**Authors:** Irma Wati Ngadimon, Angel Aledo-Serrano, Alina Arulsamy, Devi Mohan, Ching Soong Khoo, Wing Loong Cheong, Mohd. Farooq Shaikh

**Affiliations:** ^1^Neuropharmacology Research Strength, Jeffrey Cheah School of Medicine and Health Sciences, Monash University Malaysia, Subang Jaya, Malaysia; ^2^Epilepsy Program, Neurology Department, Ruber Internacional Hospital, Madrid, Spain; ^3^Global Public Health, Jeffrey Cheah School of Medicine and Health Sciences, Monash University Malaysia, Subang Jaya, Malaysia; ^4^Neurology Unit, Department of Medicine, Universiti Kebangsaan Malaysia Medical Centre, Kuala Lumpur, Malaysia; ^5^School of Pharmacy, Monash University Malaysia, Subang Jaya, Malaysia

**Keywords:** traumatic brain injury, head injury, cognitive decline, neuropsychological deficit, post-traumatic epilepsy

## Abstract

**Background:**

Post-traumatic epilepsy (PTE) is a devastating neurological outcome of traumatic brain injury (TBI), which may negatively impact the quality of life of patients with TBI, and may impose a huge socioeconomic burden. This burden may be due to long-term functional outcomes associated with PTE, particularly cognitive dysfunction. To date, the relationship between TBI and PTE remains unclear, with little known about how the effect of their link on cognitive function as well.

**Objective:**

Thus, this systematic review aimed at elucidating the relationship between PTE and cognitive impairment in adults after TBI based on available clinical studies, in hopes to aid in the development of therapeutic strategies for PTE.

**Methods:**

A systematic literature search was performed using 6 databases; MEDLINE, Embase, CINAHL, Psych INFO, Web of Science, and Cochrane to retrieve relevant clinical studies investigating the link between PTE and cognition in the context of TBI. The Newcastle-Ottawa Scale (NOS) was used to assess the methodological quality of relevant studies.

**Results:**

A total of six eligible studies were included for critical appraisal in this review after performing the inclusion and exclusion criteria, which involved 1,100 individuals, from 1996 to 2021. The selected studies were derived from the civilian and military population, with a follow-up period that ranged from 6 months to 35 years. The average quality of the involved studies was moderate (6.6, SD = 1.89). Five out of six studies found poorer cognitive performance in people with PTE, compared with those without PTE. Although the association between PTE and cognitive impairment was insignificant after controlling for specific covariates, there was a statistical trend toward significance.

**Conclusion:**

This systematic review suggests that there may be a possible link between PTE and cognitive decline in TBI patients, with the latter being reported to occur up to 35 years post injury. Variations in sample sizes, follow-up periods, and neuropsychological assessment tools may be the limitations affecting the interpretation and significance of this relationship. Therefore, future studies with standard cognitive assessment tools may be warranted to solidify the link between TBI-PTE-cognitive dysfunction, prior to the development of therapeutic strategies.

**Systematic Review Registration:** https://www.crd.york.ac.uk/prospero/display_record.php?ID=CRD42020221702, prospero identifier: CRD42020221702.

## Introduction

Traumatic brain injury (TBI) is among the leading causes of death and disability worldwide, which imposes a huge socioeconomic burden (1), with at least 10 million new cases reported each year (2, 3). TBI may be the precursor to a wide range of neurological disorders, with one of the most significantly devastating sequelae being post-traumatic epilepsy (PTE) (4–6). PTE refers to the epilepsy with unprovoked seizures that develops more than a week after TBI (7). Although the frequency may not be well known, the estimated PTE prevalence rate ranges from 1.9 to 53.3% (8, 9), depending on the age, TBI severity, and prior comorbidities. Annegers et al. (10) had earlier showed that PTE incidence ratio was only at 1.5 after mild injuries, but increased to 2.9 in moderate TBI and 17.0 in severe TBI (10), showcasing that patients with severe TBI were at a greater risk toward PTE. Moreover, a more recent study showed that veterans with penetrating TBI were at least 18 times more strongly associated with PTE development than other forms of TBI (11). PTE was deemed as the most common type of acquired epilepsy among young people, and may be closely linked to poorer functional outcomes and increased mortality (8, 9). The occurrence of PTE may impose a significant burden on patients and caregivers, mainly because of its treatment complexity (12).

Both the TBI and epilepsy elicit a wide variety of long-term consequences, independently, from physical disabilities to cognitive, emotional, and behavioral deficits, resulting in family disruptions, social restrictions, lack of earning capacity, high lifelong costs, and poor quality of life (13–15). Among them, the cognitive dysfunction may be the most well documented (16, 17). Intellectual ability, attention/concentration, processing speed, language, memory, and visual-spatial abilities are all the different domains encompassed in the term cognitive functioning (18). Thus, the functionality of these domains may be worsen in PTE, given the dual impact of the disease.

Moreover, several preclinical studies have suggested this relationship between PTE and further aggravation of cognitive impairment post-TBI. Research on mice subjected to TBI followed by the induction of seizures using electroconvulsive shock presented a worsen Barnes maze performance (learning and memory) than those with TBI only (19). In contrast, a study by Shultz et al. (20) that compared rats with or without PTE development 6 months post injury, showed no significant differences in the cognitive parameters measured by the Morris water maze. This evidence in the preclinical literature suggests that the impact of PTE on cognition may be inconclusive to date, which could be attributed to the inconsistency or variability between the PTE animal-models (spontaneous vs. induced). Hence, clinical studies on spontaneous PTE may provide better understanding on the impact of PTE on the cognition and neuropsychological performance post-TBI.

To date, there has been no systematic review to elucidate the relationship between PTE and cognitive dysfunction in the clinical context, despite the relationship of TBI and PTE or cognition being well-researched previously. Thus, this study aimed to investigate the association between PTE and cognitive dysfunction among the post-TBI population, by critical reviewing the recent publications in the clinical literature. This review may help to enlighten better therapeutic strategies to relieve cognitive dysfunction among patients with PTE and TBI, thereby improving the quality of lives of the patients, their families and caregivers.

## Methodology

### Protocol Registration

The review was conducted following the Preferred Reporting Items for Systematic Reviews and Meta-Analyses (PRISMA) 2020 guidelines (21) and was registered in the PROSPERO database (Registration number CRD42020221702), available at https://www.crd.york.ac.uk/prospero/display_record.php?ID=CRD42020221702.

### Literature Search and Selection

The literature search was conducted using six databases: Web of Science, MEDLINE, CINAHL, EMBASE, Cochrane, and PsycINFO, with articles retrieved up to March 15, 2021. The search terms utilized encompassed variations of the word post-traumatic epilepsy, traumatic brain injury, and cognitive impairment, as detailed in Supplementary Material 1. Besides that, snowballing method from the cited references was used to identify any relevant papers.

The literature selection, which was based on the PRISMA guidelines and set inclusion and exclusion criteria, was performed by two independent researchers. A third researcher was consulted to address any potential disagreements. The inclusion criteria were: (1) studies which included adult participants/population, (2) original research published in peer-reviewed journals, (3) primary studies with the following study designs: interventional and observational studies, prospective cohorts, cross-sectional and case-control design, and (4) studies confirming the presence of PTE, performed a cognitive performance assessment and reported details on the link/relationship between PTE and cognitive function, with no restriction set on the baseline cognitive function. The exclusion criteria were: (1) non-English language articles; (2) non-original research articles; (3) articles that did not investigate PTE in the context of cognitive function; and (4) studies with inadequate information to critically evaluate the strength of the results/findings.

### Data Extraction and Synthesis of Results

The study design, location of study conducted, first author and publication year, study population characteristics, assessment of PTE (seizure frequency, duration and type) and cognition, statistical analysis utilized, and significant findings pertaining to cognition in the context of PTE were extracted from each of the selected articles included in this review. It was not possible to pool the findings for a meta-analysis due to significant gaps in the measurements of cognitive function performed in the selected studies. Thus, the findings were reported qualitatively and stratified based on the principal means of cognitive assessment utilized in the studies.

### Quality Assessment of Studies

The Newcastle-Ottawa Quality Assessment Scale (NOS) was used to perform the risk of bias assessment (22). A maximum of 4 points was awarded to any study in the selection section if: (i) the study represented the PTE population; (ii) the non-PTE participants were from the TBI population; (iii) PTE was identified through a record of medical evidence; and (iv) the cognitive assessment was performed at the beginning of the study (3 points if performed within the outcome) (23). Two additional points were awarded for the comparability aspect (in this review, if the TBI severity were controlled), where an extra 1 point will be awarded for consideration of other confounders. Thus, on a scale of 0–9 points, a study may be rated as having high (7–9 points), medium (4–6 points), and low quality (0–3 points) based on the points awarded (Effect of Tobacco Smoking on the Risk of Developing Community Acquired Pneumonia: A Systematic Review and Meta-Analysis, 2019). Supplementary Material 2 contains the quality assessment of selected articles included in this review.

## Results

### Study Selection

The initial search and retrieval of articles yielded 361 articles. Twelve articles were removed for duplication and 301 articles were excluded during title and abstract screening for non-original research articles and irrelevant studies (articles that do not fit the aim of the studies and were non-clinical studies). Out of remaining 48 articles, 44 studies, that appeared to meet the inclusion criteria, were excluded for several reasons: (1) population studied was not exclusively PTE (24, 25), (2) no ascertainment of PTE where the neuropsychological outcome could only be correlated to head injury (26, 27), and (3) no statement on the association of PTE with cognition (28, 29). Therefore, the final number of eligible and relevant full-text articles included in this systematic review for critical appraisal was four clinical studies.

**Figure 1:**
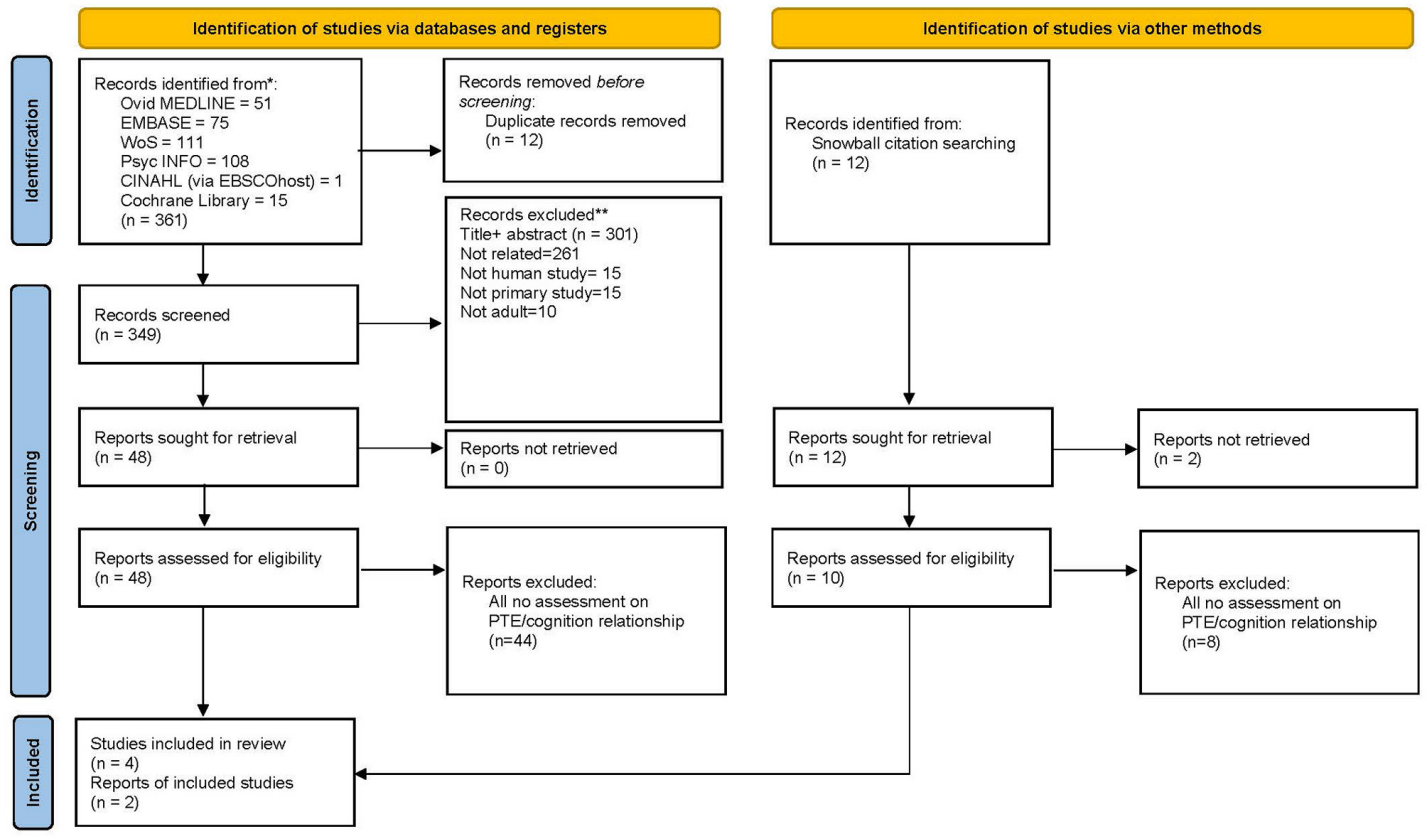
PRISMA 2020 flow diagram for new systematic reviews which included searches of databases and other sources used in this study.

As for articles retrieved through the snowballing technique, only two studies were finally included out of 12 articles identified. Thus, this brought the total number of studies included in this review to six articles (Figure 1).

### Study Characteristics

This review selected six studies to be critically appraised, which range from 1996 to 2021. These studies were predominantly from the United States (30–32), with only 2 from Italy (33, 34). Only one study was on veterans from the Vietnam War (35). All studies were observational, a mixture of prospective and retrospective studies, with 1,100 individuals in cumulative total of participants from all six studies. The age ranges of these participants were 11–79 years old, with a higher proportion of male subjects (80.1%) in comparison with female subject represented in these studies (30–34). The follow-up period ranged from 6 months to 35 years. In three of the studies, the participants were recruited before the development of PTE (31, 33, 35), but had an incidence rate of 11.9 (31), 19 (33) and 43.7% (35), respectively. These six studies assessed cognition in three ways: by using a neuropsychological psychometric tests (31, 33), by assessing global/general cognitive ability (31, 35), and by using a cognitive rating scale (30, 32, 34). The characteristics of the reviewed studies are summarized in Table 1.

**Table 1:** Characteristics of reviewed studies.

**Study**	**Country, settings and design**	**Study population (n)**	**% Epileptic vs. % non-epileptic**	**How PTE was examined**	**How cognitive function/status was ascertained**	**Cognitive outcome assessed**
Haltiner et al. (31)	USA, clinical, longitudinal cohort study	From Harborview Medical Center trauma center (210) 1. Sex distribution: more men (76%) than women (24%) 2. Mean age 28.7 (SD 11.5) years old 3. Follow-up period: 1 year 4. Cognitive status at enrolment: cognitively intact 5. Inclusion/exclusion criteria: Included if presence of depressed skull fracture, a penetrating head wound, a seizure within 24 hours of injury, cortical contusion documented by CT scan, acute intracranial hematoma (subdural, epidural, or intracerebral), or GCS; score of ≤10 on admission to ER. Excluded if age less than 16 years, non-English speaker, or a premorbid history of any of the following; seizures or on AED, a prior significant head injury, another neurological disorder, intellectual disability, a significant psychiatric disorder (e.g., schizophrenia, bipolar affective disorder), or alcoholism requiring treatment. 6. TBI category at time of injury: Moderate to Severe (GCS < 10)	Posttraumatic epilepsy (PTE) = 25 (11.9%) Late posttraumatic seizure −1 episode (LPTS) = 13 (6.19%) No late posttraumatic seizure (NLPTS) = 172 (81.9%)	-Diagnosed by a clinician based on observation or reported seizure attack. -EEG.	Halstead-Reitan Neuropsychological Test Battery: i) Verbal and nonverbal intellectual functioning (Wechsler Adult Intelligence Scale, Verbal and Performance IQ). ii) Verbal and visual-spatial memory abilities (Logical Memory and Visual Reproduction, 30-min delayed recall) iii) Motor functioning (FingerTapping with the dominant and nondominant hand) iv) Various aspects of attention, concentration, mental flexibility and speed (Trail Making Test Parts A and B, a half- page version of the Stroop Color and Word Test-Parts I and 2 and the Seashore Rhythm Test) v) Problem solving and reasoning ability (Category Test).	Cognitive decline
Mazzini et al. (33)	Italy, clinical, longitudinal cohort study	From a Rehabilitation Clinic of Veruno, Italy for postinjury rehabilitation (143) 1. Sex distribution: More men (82.52%) than women (17.48%) 2. Mean age: 32.3 (SD 15), age range 11-79 years old 3. Follow up period: ≥1 year 4. Cognitive status at baseline: Patients who emerged from coma/the vegetative state achieved cut off score of the Preliminary Neuropsychological Battery 5. Inclusion/exclusion criteria: Excluded if known to have had neurologic deficits before trauma 6. TBI category at time of injury= Severe (defined as duration of coma of ≥6 hours in the acute phase).	PTE: 27 (19%), non-PTE: 116(81%)	-Diagnosed by a clinician based on observation, anamnestic data, frequency and duration. -MRI. -EEG. -SPECT to interpret level of cerebral hypoperfusion.	i) Attention (Stroop Test, Reaction Time Test; according to the double paradigm simple and go/no-go visual RT, Digit Cancellation Test ii) Intelligence (Raven Test PM 47 colored matrices iii) Episodic Memory; according to the dichotomy of verbal/nonverbal information recall (Verbal Learning, Corsi Block Tapping Test) iv) Language (Token Test, Object naming (personal data), Phonetically cued word-fluency task, Semantic fluency task) v) Spatial Cognition (Judgment of line Orientation) vi) Perception (Tactile Form Perception)	Cognitive decline
Raymont et al. (35)	Vietnam war veterans, prospective longitudinal cohort study	Phase 3 study from the Vietnam Head Injury Study (VHIS) war veterans with head injuries (199) 1. Sex distribution: Not mentioned 2. Mean age: 58.3 (SD 2.9) 3. Follow up period: 30 to 35 years after injury 4. Cognitive status at baseline: based on previous result of AFQT (Phase 2). No significant differences of preinjury AFQT scores between subjects with and without PTE. 5. Inclusion/exclusion criteria: subjects with head injuries attended Phase 1/Phase 2 of the study, and subjects recruited through advertisements in veteran publications. 6. TBI category at time of injury= Any type of head injury was recruited. TBI category not mentioned.	PTE: 43.7% (87 out of 199 subjects)	Diagnosis based solely on semi structured interview by a neurologist experienced with the population.	i) Armed Forces Qualification Test (AFQT) ii) Wechsler Adult Intelligence Scale (WAIS).	Cognitive decline
Bushnik et al. (30)	USA, Community, prospective survey study	Drawn from 3,889 individuals in TBI Model Systems (TBIMS) National Database between 1989 and 2002 (182) 1. Sex distribution: More men (84%) 2. Mean age: Between 21–40 (56%) 3. Follow up period: 1, 2- and 5-years post-injury 4. Cognitive status at baseline: admission and discharge FIM score were comparable showing improvement from the time of admission to discharge from rehabilitation. 5. Inclusion/exclusion criteria: individuals who reported seizures within the first 2 years postinjury (LPTS group) and those who remained seizure free for the first 2 years postinjury (non-LPTS group). 6. TBI category at time of injury: Moderate to severe as indicated by time to follow command (79%)	91 LPTS (50%) 91 non-LPTS (50%) (matched pairs in the LPTS (late post-traumatic seizure) and non-LPTS groups).	Based on participants' report during interview, coded for seizure occurrence in the previous year into categories: “no seizures,” “1 seizure,” “multiple seizures,” and “un-known.”	i) Functional Independence Measure (FIM) Cognitive subscale score	Cognitive function
Kolakowsky-Hayner et al. (32)	USA, Clinical, Prospective, multi center mixed method qualitative and quantitative interview	From another longitudinal study of TBI patients admitted to Santa Clara Valley Medical Center whom reported LPTS during the first 2 years post-TBI. The patients were recontacted through TBI Model System National Database and interviewed. (25) 1. Sex distribution: More men (80%) than women (20%). 2. Mean age: 34.8 (SD 12.4), age range 20–57 years old 3. Follow up period: 5–13 years 4. Cognitive status at baseline: Not mentioned 5. Inclusion/exclusion criteria: TBI Individuals whom experienced LPTS from the previous longitudinal study (individuals with TBI from the TBI Model System National Database who were injured and reported LPTS during the first 2 years post-TBI). 6. TBI category at time of injury= Mild 16%, Moderate 32%, Severe 44%, Unknown-Sedated 8%.	All LPTS (100%) ≤1 year:28%, 1–5 year: 32%, >5 years: 40%	Based on patient's self-report to clinician	i) Craig Handicap Assessment Reporting Technique-Short Form (CHART-SF) sub-scales: Cognitive Independence	Cognitive function
Pingue et al. (34)	Italy, Clinical, retrospective observational study	From a Neurorehabilitation Unit of ICS Maugeri of Pavia, Italy (341) 1. Sex distribution: More men (78%) than women (22%) 2. Age: Mostly below 66 years of age at the time of TBI. 3. Follow up period: 6 months 4. Cognitive status at baseline: Cognitive function during admission was used as baseline. 5. Inclusion/exclusion criteria: Included if aged ≥ 18 years, diagnosis of TBI on presentation, admission to a hospital emergency department within 24 H of injury, admission within 1 month from the injury to the rehab unit to continue clinical care and rehab program, up to 6 months of observation in the rehabilitation setting. Excluded if data regarding acute care were not available, had pre-existing brain injury or other neurological disease, history of epilepsy and concurrent use of anti-seizure medication. 6. TBI category at time of injury= Mild 11.9%, Moderate 22.2%, Severe 65.9%	Early PTS (7%) Late PTS (9.4%) Early+LPTS (3%)	-Identified via medical records and classified based on time from injury into 2 classes; 1–7 days after TBI (early) vs. >7 days after TBI (late). -Physicians examined any paroxysmal clinical event described by patients or eyewitnesses that occurred during hospitalization. -Neurophysiological studies for confirmation.	i) Functional Independence Measure-Cognitive (FIM-C)	Cognitive decline

### Quality Assessment

Based on the NOS assessment tool, three studies were rated as high quality (31, 33, 35), while the other three studies were classified as medium quality (30, 32, 34). All studies had good representation of the interested population (individuals with PTE) and a clear assessment on the outcome of cognitive impairment. All studies stated that the cognitive impairment was not present prior to TBI in their subject cohort. These studies have mentioned that people with a history of neurological diseases or prior cognitive dysfunction were excluded. Four studies (66.67%) confirmed the presence of PTE through the clinical diagnosis or electroencephalogram (EEG) and five studies (83.33%) performed long-term follow-up investigations which allowed the development of neuropsychological/cognitive deficits. Four out of the six selected studies (66.67%) had an adequate follow-up of cohorts with not more than 20% withdrawal during the follow-up period.

The main study design characteristics which impacted the quality scores were the lack of selection of non-exposed cohort, as two studies did not include a control group of TBI subjects without PTE (32, 34). Another characteristic that impacted the quality of the selected articles was the lack of information/assessment on confounders, where three studies did not control for the effects of head injury severity (30, 32, 35).

### Assessment of PTE

Majority of the selected studies diagnosed PTE *via* clinical observation of involuntary movements, alterations in consciousness, or abnormal motor, or sensory function by experienced clinicians or neurologists (31, 33, 34), while some diagnosed PTE using patient-reported seizure activity through a semi-structured interview (35). The EEG monitoring was used as an adjunct diagnosis tool in some studies (31, 33).

Higher incidences of PTE were seen in the Mazzini et al. (33) study with 19% compared with the 11.9% seen in the Haltiner et al. (31) study, both at 1-year follow-up. Aligned with the other TBI study findings (4, 5, 9, 36, 37), the prevalence of PTE in both these studies were associated with the increasing injury severity and the presence and increasing frequency of neurosurgically treated subdural hematoma or intracerebral hematoma. However, all subjects in both studies had existing seizure risk factors upon enrolment. Interestingly, PTE was seen significantly more correlated with hypoperfusion in the temporal lobes, compared to those without seizures (*p* < 0.004), with lower Glasgow Coma Scale (GCS) on admission (31) or with longer coma duration (*p* < 0.007) (33).

The limitations presented in these studies in terms of PTE assessment were the knowledge transfer of previous assessment of seizures from another larger study (32, 35), and conducting follow-up seizure assessment based on the self-report method (without witness or medical report) (30, 32).

### Assessment of Cognitive Impairment

We have grouped the cognitive results from the selected studies into three main assessment methods that were utilized: neuropsychological testing, global cognitive ability, and rating scale, as depicted in Table 2.

**Table 2:** The association between post-traumatic epilepsy (PTE) and cognitive performance.

**Study**	**Quality assessment**	**Duration and frequency of seizure episode**	**Type of seizure**	**Type of cognitive assessment**	**Categorization of cognitive scores/function**	**Type of analysis**	**Results**	**Direction of association**
**THE ASSOCIATION BETWEEN PTE AND COGNITIVE (BASED ON NEUROPSYCHOLOGICAL TESTING)**								
Haltiner et al. (31)	9 (high)	Frequency: 1: 34%, 2–3: 21%, 4–9: 21%, ≥10: 24%	Focal: 23%, Generalized (with or without focal onset: 56%), Mixed: 21%	Halstead-Reitan Neuropsychological Test Battery (memory, motor, attention, concentration, mental flexibility and speed, Problem solving and reasoning ability), Halstead impairment index.	Higher scores reflect better performance on the measures memory, the Seashore Rhythm Test, and on Finger Tapping. Lower scores represent better performance on the Trail Making Test, the Stroop Color and Word Test, the Category Test, and the Halstead Impairment Index.	ANOVA (Newman-Keuls test) ANCOVA	PTE group is significantly more impaired than NLPTS group in all Halstead neuropsychological test battery and Halstead impairment index (p < 0.001). PTE group is significantly more impaired than LPTS group in all Halstead neuropsychological test battery except memory test and Halstead impairment index (*p* < 0.001). No significant differences are seen in Halstead neuropsychological test performance and Halstead impairment index at 1 year between all groups (PTE, LPTS, NLPTS). Adjusted for: i) Injury severity includes (1) the initial GCS score; (2) time to following commands (TFC); (3) CT-scan evidence of an acute subdural, epidural, or intra- cerebral hematoma; and (4) whether neurosurgery was performed to evacuate a subdural, epidural, or intracerebral hematoma, or to treat traumatic hydrocephalus. ii) demographic; age and years of education	PTE > cognitive decline than NLPTS PTE > cognitive decline than LPTS except in memory No statistically significant association after controlling the covariates
Mazzini et al. (33)	9 (high)	Duration: Complex partial seizures longer seizure duration than generalized seizures (p < 0.004).	1st seizure: Generalized (*n* = 22), complex partial (*n* = 2), simple partial (*n* = 3). Status epilepticus (*n* = 3). Recurrent seizure: similar to 1^st^ seizure except 2 patients change from generalized to simple partial.	Attention, intelligence, memory, language, spatial cognition, perception	Normal: score 0 Mildly impaired: score 1 Severely impaired: score 2	ANOVA (Spearman rank)	No significant differences are found in neuropsychological test between PTE and without PTE group at 1 year after the trauma. Adjusted for: severity of injury measured by GOS at the time of injury, duration of coma, severity of initial CT scan, and age of the patient.	No association after controlling the covariates
**THE ASSOCIATION BETWEEN PTE AND COGNITIVE (BASED ON GLOBAL COGNITIVE ABILITY SCALE)**								
Haltiner et al. (31)	8 (high)	Frequency: 1: 34%, 2–3: 21%, 4–9: 21%, ≥10: 24%	Focal: 23%, Generalized (with or without focal onset: 56%), Mixed: 21%	Wechsler Adult Intelligence Scale (WAIS, Verbal and Performance IQ)	Higher scores reflect better performance on the measures of intelligence.	ANOVA (Newman-Keuls test) ANCOVA	PTE group is significantly more impaired than NLPTS group in WAIS test (*p* < 0.001). No significant differences are seen in WAIS test performance between PTE group and LPTS group. Adjusted for: i) Injury severity includes (1) the initial GCS score; (2) time to following commands (TFC); (3) CT-scan evidence of an acute subdural, epidural, or intra- cerebral hematoma; and (4) whether neurosurgery was performed to evacuate a subdural, epidural, or intracerebral hematoma, or to treat traumatic hydrocephalus. ii) demographic; age and years of education	PTE > cognitive decline than NLPTS No significant difference in intelligence decline in both PTE and LPTS. No statistically significant association after controlling the covariates
Raymont et al. (35)	8 (high)	Mean duration of last seizure: 33 months Frequency: 2 to 10 seizures/year	Simple partial evolving to generalized: 33.3%. Generalized: 20.7%	Armed Forces Qualification Test (AFQT), WAIS	Continuous	Chi square Chi square Chi square	Significant differences of the AFQT mean score are seen between group with PTE and without PTE in in PH2 (*p* = 0.000) Significant differences of the AFQT mean score are found between group with PTE and without PTE in PH3 (*p* = 0.000). Significant differences of change in AFQT scores from preinjury to PH3 are found between group with PTE and without PTE in in PH3 (F = 4.140, df = 6, *p* = 0.000). Significant differences of the WAIS mean score are seen between group with PTE and without PTE in PH3 (*p* = 0.000). Association between the frequency of last reported seizures and PH3 AFQT score (F = 5.876, df = 6, *p* = 0.000) Association between the frequency of last reported seizures and change in AFQT scores from preinjury to PH3 (*F* = 4.140, df = 6, *p* = 0.001) Association between the type of last seizure and PH3 AFQT score (F = 6.010, df = 5, *p* = 0.000). Association between the type of last seizure and change in AFQT scores from preinjury to PH3 (F = 4.140, df = 6, *p* = 0.000). PTE is predictive of current intelligence (F = 4.102, df = 2, *p* = 0.018) and decline in AFQT score from preinjury to PH3 (F = 4.102, df = 2, *p* = 0.018)	With PTE- > cognitive decline With PTE- > cognitive decline With PTE- > cognitive decline With PTE- > cognitive decline >frequent seizure->cognitive decline > frequent seizure->cognitive decline Partial seizure evolving to generalized- >cognitive decline Partial seizure evolving to generalized ->cognitive decline
**THE ASSOCIATION BETWEEN PTE AND COGNITIVE (BASED ON RATING SCALE)**								
Bushnik et al. (30)	5 (medium)	1-year post injury = $\frac{1}{2}$ to 2/3 of individuals with End of 2^nd^ year = >75%	Not mentioned	FIM Cognitive subscale	Higher scores reflect better performance (moderately severe), to 30 (signifying death).	Repeated measure analysis of variance	LPTS group has significantly lower cognitive scores at all 3 time points than individuals in the non-LPTS group (F = 6.780; *P* < 0.01), although there are no significant differences at rehabilitation discharge. FIM Motor subscale scores do not show significant differences.	PTE- > cognitive decline
Kolakowsky-Hayner et al. (32)	4 (medium)	Seizure activity last year: 1x = 29%(*n =* 2) Multiple seizures: 71% (*n =* 5) (range 4–24 seizure events)	Not mentioned	CHART-SF sub-scales: Cognitive Independence,	CHART SF subscale score range from 0–100 (no deficit)	Descriptive	Mean = 74.84 (SD = 25.87). Range: 15–100. Score range: 0–100 (no deficit in sub-scale)	No cognitive decline
Pingue et al. (34)	6 (medium)	Early PTS: (EPTS) 7%, Late PTS (LPTS): 9.4%; Both types (early and late): 3.0% (Total: 19.4%)	Not mentioned.	Functional Independence Measure (FIM) Cognition score	Higher scores reflect better performance	Logistic regression	Presence of seizures is associated with a worse score on GCS (*p* < 0.05) and FIM (*p* < 0.01) at the end of inpatient rehabilitation. Patients with LPTS have a significantly higher risk of worse neurological outcomes than those with EPTS (*p* < 0.0001)	PTE- > cognitive decline

#### Neuropsychological Testing

Two studies which scored high methodological quality based on the NOS tool, measured cognitive performance using the established neuropsychological testing (31, 33) (Table 2). Haltiner et al. (31) used the Halstead-Reitan Neuropsychological Test Battery to determine the cognitive function of memory, motor, attention, concentration, mental flexibility, speed, problem-solving, and reasoning ability. Similarly, a study by Mazzini et al. (33) used a list of neuropsychological evaluations to assess memory, intelligence, attention, language, spatial cognition, and the perception domains.

In a study by Haltiner et al. (31), individuals with single late post-traumatic seizure (LPTS) performed slightly worse than those with no late post-traumatic seizure (NLPTS). The Haltiner et al. (31) study indicated a more significant cognitive impairment in PTE groups than the NLPTS and LPTS groups on nearly all neuropsychological measurements (*p* < 0.001), except for memory. However, when adjusted for injury severity factors, time to follow commands, CT-scan evidence of hematoma, neurosurgery procedure, and shunt placement, the significant differences in the neuropsychological assessments were no longer seen. This was also true when the demographics factors, such as age and education level, were adjusted. Similarly, the result of the psychometric analysis in the Mazzini et al. (33) study was skewed to suggest worsened cognitive functioning in the PTE group. Nonetheless, no significant difference in the cognition was observed between the PTE and non-PTE groups at 1 year after trauma.

#### Global Cognitive Ability Scale

Two high-quality studies examined cognition using global cognitive ability scales on participants, as shown in Table 2 (31, 35). Both studies reported using the Wechsler Adult Intelligence Scale (WAIS), a primary clinical instrument used to measure intelligence. Additionally, Raymont et al. (35), the only study performed on the military population, reported differences in the Armed Forces Qualification Test (38) (AFQT) results in the phase 2 (PH2) (15 years post injury) and phase 3 (PH3) (30–35 years post injury) timepoints.

The WAIS test in Haltiner et al. (31) study showed that when the TBI severity was taken into account, there were no longer significant differences in the verbal and IQ performances seen at 1-year post injury (*p* = 0.19). On the other hand, Raymont et al. (35) reported a highly significant difference in the WAIS mean score between the PTE and non-PTE groups at 30–35 years post-TBI (*p* = 0.000).

In the AFQT test reported by Raymont et al. (35), the PTE group showed a significant decline in cognition (*p* = 0.000) in their PH2 and PH3 scores than the non-PTE. More frequent seizures were associated with lower cognitive scores and a significant decline in intelligence (*F* = 5.876, df = 6, *p* < 0.001). Besides, a significant association between the type of last seizure and PH3 score (*F* = 6.010, df = 5, *p* = 0.000) and change in pre-TBI to PH3 scores were also reported (*F* = 4.140, df = 6, *p* = 0.000). Those with focal seizures with secondary generalization had the lowest PH3 scores and the most severe cognitive declined from pre-TBI to PH3. In contrast, those having focal seizures without impaired awareness had the highest scores at PH3 and the most negligible intelligence decline. In addition, those with parietal lesions (*p* = 0.001) and left insula involvement (*p* = 0.002) were more likely to report a history of seizures. The number of lobes involved was correlated with the occurrence of the complex partial seizure (*r* = 0.196, *p* = 0.006) post injury.

#### Cognitive by Uncommon Rating Scales

Three medium-quality studies reported the cognitive performance of PTE participants using less common rating scales, as in Table 2 (30, 32, 34). Bushnik et al. (30) followed 91 matched pairs of PTE and non-PTE groups at 1, 2, and 5 years post-TBI. Up to 5 years, there were no significant differences in injury severity and productivity outcomes, showing comparable cognitive improvement from the time of admission to discharge from rehabilitation. However, the Functional Independence Measure (FIM) Cognitive subscale scores showed a significant effect of seizure; the PTE group had lower cognitive scores at all 3-time points than non-PTE (*F* = 6.780; *p* < 0.01).

As for Pingue et al. (34), who retrospectively examined the clinical data of 341 adults for at least 6 months post injury, showed that at the end of the inpatient rehabilitation, the presence of seizures was linked to a lower GCS score (*p* < 0.05) and FIM (*p* < 0.01). This study had 11.9% mild TBI, 22.2% moderate, and 65.9% severe cases, with a majority (61.5%) of them having multiple site lesions, with the frontal (17.2%) and temporal lobes (13.2%) being the most affected. Early post-traumatic seizure (EPTS) was documented in 7% of cases, LPTS in 9.4%, and both EPTS and LPTS in 3% of cases. The LPTS group had a considerably higher risk of poorer neurological (*p* < 0.0001) and functional outcomes (*p* < 0.05) than EPTS group, where the risk was not significant.

Another study by Kolakowsky-Hayner et al. (32) has reported the cognitive performance scale of 25 individuals using the Craig Handicap Assessment Reporting Technique-Short Form (CHART-SF). The TBI cases in this study consisted of 16% (*n* = 4) mild, 32% (*n* = 8) moderate, 44% (*n* = 11) severe, while 8% (*n* = 2) were of unknown severity (sedated). Despite 28% of individuals having or had seizures within the last year, most experienced multiple seizures per year (range 4 to seizure events), but their mean cognitive score was still at 74.84 (SD = 25.87), which indicated no major impairment.

## Discussion

This review showed that the current literature on PTE's impact on the cognitive function among patients with TBI may still be inconclusive, with some studies indicating a strong relationship between PTE and cognitive impairment, while others showcasing no significant differences in cognitive function between PTE and non-PTE groups, even after accounting for possible confounders, such as age within each study. One of the major factors that may have led to the inconclusive evidence between PTE and cognition, may be the unstandardized neuropsychological assessment of cognition as well as the non-uniformity of patient demographics utilized between the clinical studies. Nevertheless, studies, which showed that patients with PTE recorded significant cognitive impairments, such as decline in intelligence, suggested that the TBI severity, time since PTE, PTS phase, and frequency of seizures may all contribute to the severity of the cognitive impairment in patients with PTE.

Most clinical studies in the current literature were mainly conducted to determine the association of TBI severity with cognitive deficits, as found in a literature review by McInnes et al. (39), but the similar cognitive association with PTE may still be fairly scarce. Given the pathological dynamics of PTE may differ from TBI only cases and may lead to worsen quality of life, future studies should also focus on understanding the functional outcomes in patients with PTE to determine possible therapeutic strategies for them.

Although some studies suggested a potential link between PTE and cognitive impairment (31, 33), but when the severity of the TBI was controlled, no significant differences in cognitive performance was observed between the PTE and non-PTE groups. This suggest that any cognitive impairment witnessed in these studies may be solely catered to the impact of TBI itself and not PTE. In fact, most of the selected studies had patients who were majorly diagnosed with severe TBI, suggesting that the cognitive decline witnessed may be driven by the severity of the TBI rather than the development of PTE. However, it should be noted that PTE development were more strongly evident in those with more severe TBI, whereby worsen GCS score was recorded for groups that developed PTE compared with groups that were labeled with only one late seizure episode (LPTS) or no seizure at all (NLPTS), and cognitive performance between them was shown to be similar. The higher prevalence of PTE in severe TBI was supported by the evidence in the past literature (9, 10). This suggest that the interplay between injury severity and presence of seizure may not govern the cognition any differently as their independent influence on cognition. Although, one study suggested that at 1 year post injury, this interplay may be more evident, suggesting that the long-term impact of seizures on the neuronal activity and damage, in collaboration with the initial neuronal damage post-TBI may surface greater cognitive deficits and neuropsychological morbidity. However, future studies may be required to support this theory.

Particularly since these selected studies may have their limitations which may have confounded their results. One of the limitation was the very low incidence rate of PTE in some studies, Haltiner et al. (31) reported only 11.9% of PTE incidence among patients with TBI while Mazzini et al. (33) reported a slightly higher incidence rate of 19%, both of which may be too low and may have impeded the statistical significance of the neuropsychological findings. In addition, the neuropsychological tests performed in these studies were skewed, whereby in Haltiner et al. (31) study, only 44% of patients with PTE were tested compared with the larger fraction of patients with NLPTS and LPTS who were tested on the same neuropsychological evaluation, leading to missing outcome measures and possible bias interpretations. Another limitation was the low and varied occurrence of status epilepticus (SE) or refractory epilepsy at the 1-year follow-up post-TBI; null in Haltiner et al. (31) and *n* = 3 in Mazzini et al. (33), which were often associated with worsen functional outcomes (40). It is essential to evaluate SE and refractory epilepsy in the context of PTE since they may lead to significant neurological deterioration (41, 42). In some studies, PTE was assessed based on patient's self-reportation of seizures (30, 32), which may result in incidences of under-reporting or inaccurate/bias reporting as well. While the wide age range of patient recruitment in Mazzini's study may serve as another limitation/confounding factor, the study performed a multivariate analysis which minimized or negated the effects of age on the neurobehavioral and functional outcomes (33). Additionally, a study with a short duration of less than a year (34) may impact result interpretation, since PTE and cognitive impairment usually occurs more chronically post-TBI and the time of development may vary between patients.

Thus, a chronic studies may be more relevant, such as that performed by Raymont et al. (35), which reported a high prevalence of PTE (43.7%) in those who had sustained a TBI for over 30–35 years. In support, Bushnik et al. (30) found significantly lower cognitive scores in the LPTS individuals compared to patients with TBI alone, while Pingue et al. (34) found that LPTS individuals had worsen cognitive subscale scores than those with EPTS at the end of the 6-month inpatient rehabilitation, suggesting that long-term seizures were more detrimental to cognitive function that those with early seizure onset post-TBI. However, studies on chronic stages post-TBI may need to account for the possible effects of aging on cognition that may influence PTE's relationship with cognitive impairment (43).

Interestingly, Raymont and his colleagues concluded that PTE may be predictive of current intelligence, with a decline in IQ scores seen in military patients with PTE (35). It was worth noting that the chronic follow-up time showed that patients with increasing episodes of PTE annually (i.e., frequency of 2–10 seizures per year) were associated with a higher decline in a full-scale IQ test and intelligence (35). Furthermore, individuals with focal seizures evolving to bilateral tonic-clonic seizures had the most significant cognitive decline, suggesting the influence of type and severity of seizures on cognitive functionality.

The pattern of neurocognitive deficits may have originated from the frontal or temporal lobes, which were common place of development of seizure foci in patients with PTE (16, 44, 45). Due to the neuroanatomical location, the frontal lobes (specifically the orbitofrontal region) and temporal lobes may be particularly vulnerable to TBI lesions as well (46, 47). As clinically evidenced in this review (31, 33, 34), most PTE patients with frontal and temporal lesions reported having experienced impairment in memory, language, attention, processing speed, and executive functions (mental flexibility, problem-solving and reasoning ability) (47, 48). These findings suggest that targeting the pathology within the frontal and temporal lobes post-TBI, may prevent the PTE development and subsequent cognitive decline. The pathology of PTE's impact on cognition may also extend or propagate to other areas of the brain, such as parietal lobe (35), depending on the type of TBI/injury lesions.

Instead of investigating the pathology in the brain lesions areas, future studies should also focus on determining the causal and pathophysiological relationship of PTE and cognitive impairment *via* blood or biomarkers and neuroimaging techniques. There have been some robust biomarkers of epilepsy that were suggested to correlate with the pathological progression of cognition (49, 50). For example, the blood-based biomarkers (inflammatory markers), such as interleukin-6 (IL-6) and high mobility group box 1 (HMGB1) have been shown to play a role in epilepsy progression and cognitive impairment mechanism (51–53). However, these epilepsy biomarkers may still be in the early phase of the research curve (54–56). There may still be insufficient understanding on the multidimensional cascades of TBI leading to the current ineffective treatment and prevention strategies of post-TBI functional outcomes (57).

Some other strategies to lessen the impact of PTE on cognition may include controlling the severity and frequency of seizures post-TBI. Optimal seizure control may have a positive impact on the cognitive function and quality of life of patients with PTE (15, 32, 58). Consequently, cognitive screening in new-onset epilepsies post-TBI should become a standard practice in post-injury care to provide effective therapies for early seizure control, thereby preventing or slowing the cognitive decline in later years. Information about the patient's cognitive state at the onset of epilepsy and before the initiation of treatment may enable healthcare providers to assess the disease evolution and associate the possible changes to either the effectiveness of treatment, the side effects of antiseizure medications, and the dynamics of the underlying disease post injury. Both the early detection of cognitive dysfunction and optimal seizure control may have a positive impact on a patient's social function, and may reduce caregivers burden and holistically, thereby improving quality of life of patients and caregivers alike (12, 16, 59). Follow-up assessments of new-onset epilepsies post-TBI, as well as those on epilepsy treatment in future studies, may yield more prominent findings. Besides that, more assessment indicators of cognitive comorbidity should be performed in future studies, since the spectrum of cognition may be too broad to be captured by just a single type of cognitive or neuropsychological assessment (60). Furthermore, the epileptogenesis of PTE takes months to years to manifest and only in some patients with TBI, hence increasing the number of chronic investigation studies will be more beneficial to clearly witness the adverse effects of PTE, particularly on cognition.

## Conclusion

This review found that the association between PTE and cognitive function among patients with TBI may be inconclusive to date, due to scarcity of the literature and significant limitations in the current literature, particularly in the non-uniformity and variations in the study designs, neuropsychological performance evaluation methods, and follow-up periods. People with TBI and PTE were reported to face the double-barreled disadvantages on cognition, which was evident up to 35 years of post injury. Therefore, more explicit treatment strategies for optimal seizure control and more transparent assessment guidelines may be required for individuals affected by PTE to manage and respond to these ongoing cognitive challenges. Further investigations may be required to narrow the knowledge gap in the etiology and pathology of neuropsychological decline in PTE individuals to provide the most effective therapeutic strategy, which may help patients with PTE to lead a good quality of life.

## Data Availability Statement

The original contributions presented in the study are included in the article/Supplementary Material, further inquiries can be directed to the corresponding author/s.

## Author Contributions

IN: conceptualization, data collection, investigation, literature retrieval, and writing—original draft. MS: conceptualization and supervision. AA-S: investigation and writing—review and editing. AA, CK, and WC: writing—review and editing. DM: supervision and writing—review and editing. All authors contributed to the article and approved the submitted version.

## Funding

The authors were supported by Monash University Malaysia-School of Medicine Strategic Grant 2021.

## Conflict of Interest

The authors declare that the research was conducted in the absence of any commercial or financial relationships that could be construed as a potential conflict of interest.

## Publisher's Note

All claims expressed in this article are solely those of the authors and do not necessarily represent those of their affiliated organizations, or those of the publisher, the editors and the reviewers. Any product that may be evaluated in this article, or claim that may be made by its manufacturer, is not guaranteed or endorsed by the publisher.
