# Supplementary material for: An Interplay Between Post-Traumatic Epilepsy and Associated Cognitive Decline: A Systematic Review
Source: Front Neurol. 2022 Feb 24;13:827571. doi: 10.3389/fneur.2022.827571 (PMC8908100; doi:10.3389/fneur.2022.827571)
Supplement: Supplementary file 1 [file Data_Sheet_1.DOCX]

**Supplementary File 1: Search strategy (all databases)**

**Ovid MEDLINE(R) <1946 to March 15, 2021>**

1 post*traumatic epilepsy.mp.

2 exp Epilepsy, Post-Traumatic/

3 post*traumatic seizure.mp.

4 late post*traumatic seizure.mp.

5 concussive convulsion*.mp.

6 1 or 2 or 3 or 4 or 5

7 Traumatic brain injury.mp. or exp Brain Injuries, Traumatic/

8 exp Head Injuries, Penetrating/ or exp Head Injuries, Closed/ or head injur*.mp.

9 head trauma.mp. or exp Craniocerebral Trauma/

10 exp Brain Injuries/ or brain injur*.mp.

11 brain trauma.mp.

12 brain damage.mp.

13 exp Brain Concussion/ or concussion.mp.

14 cranial trauma.mp.

15 traumatic encephalopathy*.mp.

16 7 or 8 or 9 or 10 or 11 or 12 or 13 or 14 or 15

17 cognitive impairment.mp. or exp Cognitive Dysfunction/

18 cognitive dysfunction.mp.

19 cognitive outcome.mp.

20 cognitive deficit.mp.

21 cognitive decline.mp.

22 cognitive change*.mp.

23 cognitive deteriorate*.mp.

24 cognitive performance.mp.

25 cognitive test*.mp.

26 cognitive function.mp.

27 exp Cognition/ or cognition.mp.

28 intellectual disability.mp. or exp Intellectual Disability/

29 neuropsychological assessment.mp.

30 neuropsychological outcome.mp.

31 neuropsychological test.mp. or exp Neuropsychological Tests/

32 neuropsychological measure.mp.

33 exp Neuropsychology/ or neuropsychology*.mp.

34 neurocognitive.mp.

35 17 or 18 or 19 or 20 or 21 or 22 or 23 or 24 or 25 or 26 or 27 or 28 or 29 or 30 or 31 or 32 or 33 or 34

36 6 and 16 and 35

37 limit 36 to (english language and humans and "all adult (19 plus years)")

**Embase Classic+Embase (Ovid) <1947 to 2021 March 15>**

1 exp traumatic epilepsy/ or post*traumatic epilepsy.mp.

2 exp seizure/ or post*traumatic seizure.mp.

3 late post-traumatic seizure.mp.

4 concussive convulsion*.mp. or exp brain concussion/

5 1 or 2 or 3 or 4

6 Traumatic brain injury.mp. or exp traumatic brain injury/

7 exp head injury/ or head injur*.mp.

8 head trauma.mp.

9 exp brain injury/ or brain injur*.mp.

10 brain damage.mp. or exp brain damage/

11 brain trauma.mp. or exp traumatic brain injury/

12 exp concussion/ or concussion.mp.

13 cranial trauma.mp.

14 exp chronic traumatic encephalopathy/ or traumatic encephalopathy*.mp.

15 6 or 7 or 8 or 9 or 10 or 11 or 12 or 13 or 14

16 cognitive impairment.mp. or exp cognitive defect/

17 cognitive dysfunction.mp.

18 cognitive outcome.mp.

19 cognitive deficit.mp.

20 cognitive decline.mp.

21 cognitive change*.mp.

22 exp mental deterioration/ or cognitive deteriorat*.mp.

23 cognitive performance.mp.

24 cognitive test*.mp.

25 cognitive function.mp. or cognition/

26 cognition.mp. or exp cognition assessment/

27 intellectual disability.mp. or exp intellectual impairment/

28 neuropsychological assessment.mp.

29 neuropsychological outcome.mp.

30 neuropsychological test.mp. or exp neuropsychological test/

31 neuropsychological measure.mp.

32 exp neuropsychology/ or neuropsychology*.mp.

33 neurocognitive.mp.

34 16 or 17 or 18 or 19 or 20 or 21 or 22 or 23 or 24 or 25 or 26 or 27 or 28 or 29 or 30 or 31 or 32 or 33

35 5 and 15 and 34

36 limit 35 to (english language and adult <18 to 64 years> and "humans only (removes records about animals)")

37 limit 36 to (human and english language and exclude medline journals and embase and adult <18 to 64 years>)

**Web of Science Core Collection *Indexes=SCI-EXPANDED, SSCI Timespan=All years***

1 TS=(post*traumatic epilepsy)

2 TS=(post*traumatic seizure)

3 TS=(late post-traumatic seizure)

4 TS=(concussive convulsion*)

5 #4 OR #3 OR #2 OR #1

6 TS=(Traumatic brain injury) OR TS=(head injur*) OR TS=(head trauma) OR TS=(brain injur*) OR TS=(brain trauma) OR TS=(brain damage) OR TS=(concussion) OR TS=(cranial trauma) OR TS=(traumatic encephalopathy*)

7 TS=(cognitive impairment) OR TS=(cognitive dysfunction) OR TS=(cognitive outcome) OR TS=(cognitive deficit) OR TS=(cognitive decline) OR TS=(cognitive change*) OR TS=(cognitive deteriorate*) OR TS=(cognitive performance) OR TS=(cognitive test*) OR TS=(cognitive function) OR TS=(cognition)

8 TS=(intellectual disability) OR TS=(neuropsychological assessment) OR TS=(neuropsychological outcome) OR TS=(neuropsychological test) OR TS=(neuropsychological measure) OR TS=(neuropsychology*) OR TS=(neurocognitive)

9 #8 OR #7

10 #9 AND #6 AND #5

11 #9 AND #6 AND #5

Refined by: [excluding] LANGUAGES: ( RUSSIAN )

**APA PsycInfo (Ovid) <1806 to March Week 2 2021>**

1 exp Epilepsy/ or post*traumatic epilepsy.mp.

2 exp Epileptic Seizures/ or post*traumatic seizure.mp.

3 late post-traumatic seizure.mp.

4 concussive convulsion*.mp.

5 1 or 2 or 3 or 4

6 Traumatic brain injury.mp. or exp Traumatic Brain Injury/

7 head injury.mp. or exp Head Injuries/

8 exp Trauma/ or head trauma.mp.

9 brain injur*.mp.

10 brain trauma.mp.

11 brain damage.mp. or exp Brain Damage/

12 exp Brain Concussion/ or concussion.mp.

13 cranial trauma.mp.

14 exp Encephalopathies/ or traumatic encephalopathy*.mp.

15 6 or 7 or 8 or 9 or 10 or 11 or 12 or 13 or 14

16 cognitive impairment.mp. or exp Cognitive Impairment/

17 cognitive dysfunction.mp.

18 exp Cognitive Ability/ or cognitive outcome.mp.

19 cognitive deficit.mp.

20 cognitive decline.mp. or exp Mild Cognitive Impairment/

21 cognitive change*.mp.

22 cognitive deteriorat*.mp. [mp=title, abstract, heading word, table of contents, key concepts, original title, tests & measures, mesh]

23 cognitive performance.mp.

24 exp Cognitive Assessment/ or cognitive test*.mp.

25 cognitive function.mp.

26 exp Cognition/ or cognition.mp.

27 intellectual disability.mp.

28 neuropsychological assessment.mp. or exp Neuropsychological Assessment/

29 neuropsychological outcome.mp.

30 neuropsychological test.mp.

31 exp Executive Function/ or neuropsychological measure.mp.

32 exp Neuropsychology/ or neuropsychology*.mp.

33 exp Neurocognition/ or neurocognitive.mp.

34 16 or 17 or 18 or 19 or 20 or 21 or 22 or 23 or 24 or 25 or 26 or 27 or 28 or 29 or 30 or 31 or 32 or 33

35 5 and 15 and 34

36 limit 35 to ("300 adulthood <age 18 yrs and older>" and english and human)

**CINAHL Plus (via EBSCO Host) <1966 to 2021>**

| S23 | S5 AND S15 AND S22 |  |  |
| --- | --- | --- | --- |
| S22 | S16 OR S17 OR S18 OR S19 OR S20 OR S21 |  |  |
| S21 | "( intellectual disability or mental retardation or learning disability or developmental disability or learning disabilities ) OR ( neuropsychological assessment or evaluation ) OR neuropsychological outcome OR neuropsychological test OR neuropsychological measure OR neuropsychology* OR neurocognitive" OR (MH "Neuropsychological Tests+") OR (MH "Intellectual Disability+") |  |  |
| S20 | (MH "Cognition+") OR "cognitive deteriorat* OR cognitive performance OR cognitive test* OR cognitive function OR cognition" |  |  |
| S19 | "cognitive outcome OR cognitive deficit OR cognitive decline OR cognitive change*" |  |  |
| S18 | "cognitive outcome OR cognitive deficit OR cognitive decline OR cognitive change*" |  |  |
| S17 | "cognitive dysfunction" OR (MH "Cognition Disorders+") |  |  |
| S16 | "cognitive impairment" OR (MH "Mild Cognitive Impairment") |  |  |
| S15 | S6 OR S7 OR S8 OR S9 OR S10 OR S11 OR S12 OR S13 |  |  |
| S14 | S6 OR S7 OR S8 OR S9 OR S10 OR S11 OR S12 OR S13 |  |  |
| S13 | "(concussion or mild traumatic brain injury or mild tbi or mtbi ) OR cranial trauma OR traumatic encephalopathy*" |  |  |
| S12 | "(concussion or mild traumatic brain injury or mild tbi or mtbi ) OR cranial trauma OR traumatic encephalopathy*" |  |  |
| S11 | "brain damage" |  |  |
| S10 | "brain trauma" |  |  |
| S9 | (MH "Brain Injuries") OR "brain injur*" |  |  |
| S8 | "head trauma" |  |  |
| S7 | (MH "Head Injuries+") OR "head injury" |  |  |
| S6 | "Traumatic brain injury" OR (MH "Brain Injuries+") |  |  |
| S5 | S1 OR S2 OR S3 OR S4 |  |  |
| S4 | "late post-traumatic seizure OR concussive convulsion*" |  |  |
| S3 | "late post-traumatic seizure OR concussive convulsion*" |  |  |
| S2 | "post*traumatic seizure" |  |  |
| S1 | (MH "Epilepsy, Post-Traumatic") OR "post*traumatic epilepsy" |  |  |

**Cochrane <commencement to 15^th^ March 2021>**

| 1 | Post-traumatic epilepsy |
| --- | --- |
| 2 | Post-traumatic seizure |
| 3 | late post-traumatic seizure |
| 4 | concussive convulsion |
| 5 | OR 1 - 4 |
| 6 | Traumatic brain injury |
| 7 | head injury |
| 8 | head trauma |
| 9 | brain injury |
| 10 | brain trauma |
| 11 | brain damage |
| 12 | concussion |
| 13 | cranial trauma |
| 14 | traumatic encephalopathy |
| 15 | OR 6-14 |
| 16 | cognitive impairment |
| 17 | cognitive dysfunction |
| 18 | cognitive outcome |
| 19 | cognitive deficit |
| 20 | cognitive decline |
| 21 | cognitive change |
| 22 | cognitive deteriorate |
| 23 | cognitive performance |
| 24 | cognitive test |
| 25 | cognitive function |
| 26 | cognition |
| 27 | intellectual disability |
| 28 | neuropsychological assessment |
| 29 | neuropsychological outcome |
| 30 | neuropsychological test |
| 31 | neuropsychological measure |
| 32 | neuropsychology |
| 33 | neurocognitive |
| 34 | OR 16 - 33 |
| 35 | 5 AND 15 AND 34 |
| 36 | 35 Limit to (Human, Adult, English only) |
