# Supplementary material for: An Interplay Between Post-Traumatic Epilepsy and Associated Cognitive Decline: A Systematic Review
Source: Front Neurol. 2022 Feb 24;13:827571. doi: 10.3389/fneur.2022.827571 (PMC8908100; doi:10.3389/fneur.2022.827571)
Supplement: Supplementary file 2 [file Table_1.DOCX]

**Supplementary File 2**

**Table S1. Quality assessment for all studies using Newcastle Ottawa Scale**

| Study | Selection | | | | Comparability | | Outcome | | | Score | Quality |
| --- | --- | --- | --- | --- | --- | --- | --- | --- | --- | --- | --- |
|  | Representativeness of the exposed cohort | Selection of the non-exposed cohort | Ascertainment of exposure | Demonstration that outcome of interest was not present at start of study | Comparability of cohorts on the basis of the design or analysis | | Assessment of outcome | Was follow-up long enough for outcomes to occur | Adequacy of follow up of cohorts |  |  |
|  |  |  |  |  | Main factor | Additional factor |  |  |  |  |  |
| Haltiner et al.^29^ | / | / | / | / | / | / | / | / | / | 9/9 | High |
| Mazzini et al.^31^ | / | / | / | / | / | / | / | / | / | 9/9 | High |
| Raymont et al.^33^ | / | / | / | / | 0 | 0 | / | / | / | 7/9 | High |
| Bushnik et al.^28^ | / | / | 0 | / | 0 | 0 | / | / | 0 | 5/9 | Medium |
| Kolakowsky-Hayner et al.^30^ | / | 0 | 0 | / | 0 | 0 | / | / | 0 | 4/9 | Medium |
| Pingue et al.^32^ | / | 0 | / | / | / | 0 | / | 0 | / | 6/9 | Medium |
